# Nuances in visual rehabilitation after pituitary surgery

**DOI:** 10.1093/noajnl/vdae087

**Published:** 2025-01-02

**Authors:** Saksham Gupta, Wenya Linda Bi

**Affiliations:** Center for Skull Base and Pituitary Surgery, Department of Neurosurgery, Brigham and Women’s Hospital, Harvard Medical School, Boston, MA, USA; Center for Skull Base and Pituitary Surgery, Department of Neurosurgery, Brigham and Women’s Hospital, Harvard Medical School, Boston, MA, USA

**Keywords:** vision loss, diplopia, pituitary surgery, optic nerve injury

## Abstract

**Background:**

Patients with pituitary lesions often present with visual deficits attributable to mass effect along the optic pathway or on the cranial nerves controlling extraocular muscles. While symptoms often improve after treatment, persistent symptoms negatively impact quality of life.

**Methods:**

We reviewed the literature on emerging concepts in visual monitoring and recovery during and after pituitary surgery.

**Results:**

Rigorous preoperative laboratory testing, neuro-ophthalmologic examination, and imaging abet planning of safe surgery. Intraoperative visual evoked potentials may provide an adjunct to monitor impending damage to vision during surgery, particularly for recurrent tumors that may have scarred onto visual structures. Treatment of persistent visual deficits depends on the physiological cause of the deficit, the duration of symptoms, and the degree and pace of spontaneous recovery. Management ranges from observation to corrective oculoplastic surgery, often in the context of a multidisciplinary team. There are several gaps in knowledge on the reasons why visual deficits remain after adequate decompression during surgery, and further study will reveal new therapies and devices.

**Conclusion:**

Postoperative visual deficits following pituitary surgery can cause a considerable reduction in quality of life. Multidisciplinary neurosurgery, neuro-ophthalmologic, and neuro-endocrine teams should work in concert to provide an individualized approach for each patient.

**Key Points**

1. Patients may experience visual deficits from tumor invasion or compression of the optic apparatus or the cranial nerves that control extraocular movement.
2. Treatments ranging from short-term corticosteroids to corrective oculoplastic surgery that improve persistent postoperative visual deficits from pituitary lesions and iatrogenic injuries during pituitary surgery.

**Importance of Study**

Despite advances in surgery for pituitary tumors, a subset of patients have persistent visual symptoms after treatment. This study reviews adjunctive therapies for persistent visual symptoms and deficits for pituitary surgeons.

Pituitary lesions can cause visual symptoms that limit function and quality of life, particularly when they have suprasellar or cavernous sinus extension. It is estimated that 20%–40% of patients with pituitary tumors present with visual symptoms and deficits, though tumors are increasingly found incidentally due to the routine availability and deployment of imaging.^1–4^ Approximately 2%–12% of patients present with apoplexy, a clinical syndrome of acute headache and ophthalmoplegia that results from intratumoral hemorrhage or ischemic necrosis, most often of a pituitary adenoma.^5^ Apoplexy necessitates an urgent evaluation and consideration of corticosteroids, though emerging evidence suggests that surgery can safely be performed 1–2 weeks after the apoplexy event to allow inflammation to subside.^6^ On the other end of the timescale spectrum, chronic visual deficits from pituitary lesions can remain underreported or misattributed to aging or other medical conditions by patients, despite their impact on quality of life.^7–9^ Less commonly, iatrogenic injury during pituitary surgery leads to new deficits, which can be transient or permanent.^10^ Visual deterioration can also occur in a delayed fashion after pituitary surgery due to scarring near the optic apparatus.^11^

Visual impairment contributes to a considerable decline in patients’ quality of life, and it is important to consider the underlying physiological disturbances that drive each individual’s impairment.^12,13^ In this narrative review, we describe the causes of visual impairment after pituitary surgery and discuss emerging techniques in the management of visual deficits to guide the neurosurgeon to understand and tailor treatment options for each patient.

## Patterns of Visual Deficits

**Figure 1.**
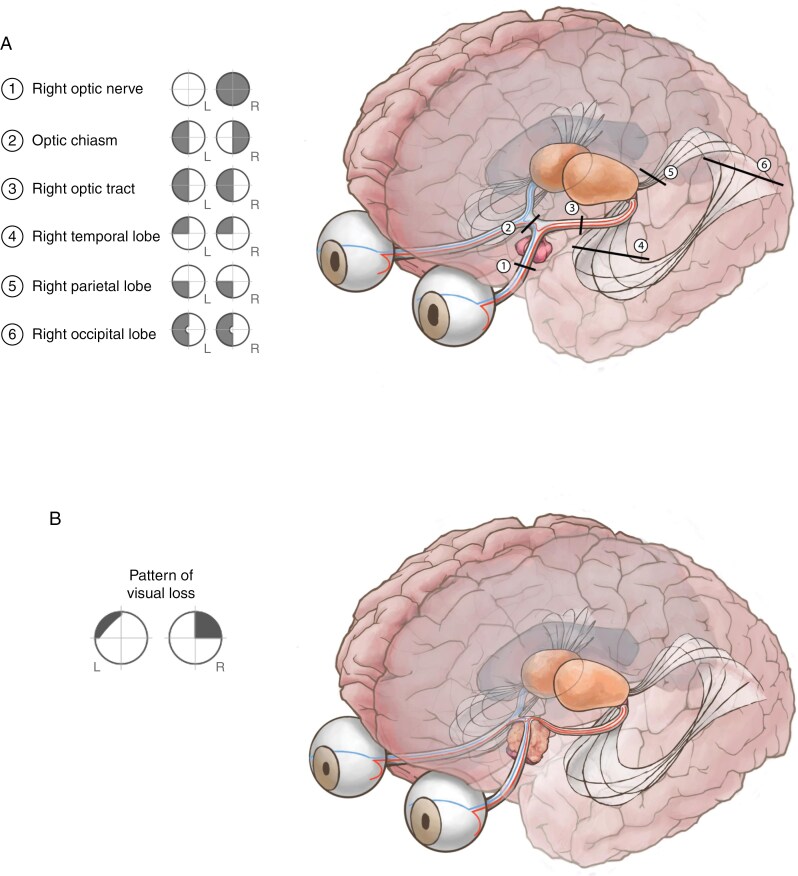
(A-B) The anterior optic pathway can be compressed along several points by suprasellar pathologies (B). A representative image and visual deficit pattern are represented for a pituitary tumor with suprasellar extension that compresses the optic chiasm (B).

The neuro-ophthalmologic exam plays a critical role in understanding the anatomic drivers of the visual deficit, which can inform surgery. Pituitary tumors causing optic nerve or chiasmal compression manifest with a range of symptoms depending on the chronicity and anatomic localization of the compression (Figure 1).^14^ Acquired color vision deficits from extrinsic optic nerve compression present as red-green color blindness and are associated with decreased visual acuity.^15^ Loss of color discrimination is common, with over half of patients presenting with partial or complete color blindness across several cohort studies.^16,17^

Pituitary tumors compressing the optic chiasm can cause partial to complete bitemporal hemianopsia due to impaired function of the nasal retinal crossing fibers.^18,19^ Severe bitemporal hemianopsia may also cause loss of stereopsis or diplopia due to the hemifield slide phenomenon, when there is insufficient overlap in the visual fields to produce a unified representation of the visual field.^20^ Chiasmal compression from pituitary tumors may also rarely cause hemihypokinesia, where one half (usually the temporal half) of the retina has an afferent pupillary defect. There are several perimetry methods to test visual fields, including the Goldmann perimetry test, which assesses acuity up to 90°C in the temporal hemifield and 60°C in the nasal hemifield, and automatic perimetry tests, which assesses up to 30°C from central vision.^14^

Cavernous sinus invasion by tumor typically produces diplopia due to cranial nerve palsies that limit ocular movement. Oculomotor nerve weakness is the most common cranial nerve deficit from pituitary tumors, followed by abducens nerve and trochlear nerve palsies.^21,22^ Ptosis is the most common presenting symptom among patients with oculomotor palsy attributable to compression from a pituitary tumor. Acute cranial nerve compression syndromes ensue from events such as apoplexy.^23^

## Preoperative Evaluation for Pituitary Lesions

A battery of tests is available to assess the hypothalamic-pituitary axis and critical neurologic and neurovascular structures near the sella. Comprehensive evaluation of hormone function establishes a necessary baseline prior to treatment and may lead to endocrinologic correction prior to surgery. Thin-cut computed tomography (CT) with possible CT angiography (CTA) elucidates nasal and sphenoid sinus anatomy and maps the location of the internal carotid arteries relative to the tumor. Preoperative magnetic resonance imaging improves the detection of optic pathways and pituitary displacement for large tumors.^24^ For smaller tumors, such as functional corticotroph microadenomas, high-sensitivity magnetic resonance imaging may improve the visualization of previously undetectable tumors.^25^

Patients with tumors abutting, displacing, or infiltrating visual structures often undergo formal ophthalmologic evaluation. Useful tests include optical coherence tomography (OCT), a noninvasive method to visualize the thickness of each retinal layer, including the retinal nerve fiber layer (RNFL) adjacent to the optic disc and ganglion cell layer (GCL). Persistent compression on the optic nerve or chiasm can cause retrograde axonal injury, thinning the RNFL and GCL as well as atrophying the retinal ganglion cell bodies, often prior to the onset of perceptible symptoms.^26–29^ Both RNFL and GCL thinning have been associated with delayed or incomplete postoperative visual recovery.^14^ In the appropriate clinical context, OCT offers a fast and noninvasive tool for screening and pre-operative counseling regarding return of visual function.^30,31^

Selection of the operative strategy depends on the origin of the tumor, cadence of symptoms, relative anatomic location to critical neurovascular structures such as the carotid artery (internal carotid arteries) and optic chiasm, and the personal experience and expertise of the surgical team. For example, a prefixed, neutral, or postfixed location of the optic chiasm relative to the pituitary gland noted on preoperative imaging influences the operative approach to minimize traction injury against the optic nerve and chiasm. The sphenoid pneumatization variation also helps dictate the optimal operative approach, particularly for the poorly pneumatized conchal pattern.

When a progressive visual deficit is present, surgery is typically performed within 1–2 weeks. Patients with preoperative optic nerve, optic chiasm, or cavernous sinus compression may benefit from temporary corticosteroids as they await surgery, though this should be used selectively since their use will impair the assessment of the hypothalamic-pituitary axis postoperatively.^32^ Apoplexy presents a unique case given the acuity of symptoms and the pro-inflammatory state caused by hemorrhage. Emergent surgery was classically the standard of care for pituitary apoplexy, but recent evidence on the benefit of early surgery is conflicting and suggests that initiating corticosteroids and waiting to perform the operation within 1–2 weeks may have equivalent rates of visual recovery for certain patients.^33–36^

Visual evoked potentials are a neurophysiological modality to monitor the optic apparatus and were first applied to pituitary surgery in the 1970s.^37,38^ Reductions in intraoperative VEP amplitude can be predictive of postoperative visual field or acuity loss across both transcranial and transsphenoidal operations that may involve manipulation of the optic nerves or chiasm.^39–43^ However, the adoption of visual evoked potentials has been limited by their variable accuracy, which may be confounded by anesthetic regimens and techniques.^42^ The oculomotor, trochlear, and abducens nerves can be monitored using electromyography (EMG) in select cases, such as a giant adenoma invading the cavernous sinus or a giant craniopharyngioma that extends posteriorly into the interpeduncular cistern.^44,45^

## Postoperative Management of Visual Impairments

Visual symptoms resolve for 70%–95% of patients postoperatively when patients undergo evaluation and surgery without delay in experienced centers.^46–49^ Visual field deficits typically recover within weeks of surgery and can continue to improve even after 3 years.^50^ Similarly, cranial nerve palsies often improve in 1–2 weeks following surgery, especially if preoperative symptoms were of a short duration.^51^ When available, OCT can help delineate the pace of recovery since patients with preoperative RNFL and GCL thinning typically have slower visual recovery.^52^ The degree and rate of improvement can be lower in low-resource settings, where patients present with advanced disease and surgeons may have lower-quality equipment to evaluate patients and perform surgery.^53,54^ After surgery, patients should also be counseled about visual fatigue and may benefit from available online tools that help manage fatigue symptoms.^55^ Poor social support further negatively affect quality of life for patients with visual impairment, and construct of a social network contributes to the recovery process.^56,57^

Adjuncts are available for patients who experience partial resolution or no improvement after surgery (Table 1). Awareness of the range of nonoperative and operative options available to improve vision recovery helps patient counseling and referrals to the appropriate medical resource.

**Table 1.** Causes and Treatments for Common Visual Deficits Attributable to Pituitary Tumors

Deficit	Anatomic cause	Treatment options
Loss of color	*Optic nerve*	Optic filter glasses^58,59^ Optic filter contact lenses^60^,^*^
Loss of acuity	*Optic nerve/chiasm*	Corrective lenses
Visual field deficit	*Optic nerve/chiasm*	Restorative rehabilitation^61,62^ Compensatory rehabilitation^59^ Visual field expansion prism glasses^,^^63^^*^
Diplopia	*Optic chiasm (hemifield slide)*	Corrective prism glasses^20^ Compensatory typoscope^6,4,^*^^
	*Oculomotor nerve*	Pre/postoperative steroids^64^ Monocular eye occlusion (ie, eye patch)^65^ Corrective prism glasses^65^ Botulinum toxin to lateral rectus^65^ Corrective surgery a. Recession of lateral rectus/resection of medial rectus^6,7^ b. Superior oblique transposition (in addition to 3a)^65,67^ c. Additional advanced surgical techniques^65^
	*Trochlear nerve*	Pre/postoperative steroids Monocular eye occlusion (ie, eye patch) Corrective prism glasses^66^ Botulinum toxin to inferior rectus^64,67^ Corrective surgery a. Inferior oblique myectomy/recession^68^ b. Superior rectus/contralateral inferior rectus recession^69^ c. Additional advanced surgical techniques^70,68^
	*Abducens nerve*	Pre/postoperative steroids^71^ Monocular eye occlusion (ie, eye patch)^7,4^ Corrective prism glasses^7,4^ Botulinum toxin to the medial rectus^72^ Corrective surgery a. Lateral rectus resection and medial rectus recession^22,7,4^ b. Additional advanced surgical techniques^7,4^

^*^Preclinical.

### Color Blindness

Patients with optic nerve damage are generally more likely to have red-green color blindness rather than blue-yellow color blindness, while the converse is true for patients with outer retinal damage. Color blindness can be tracked peri-operatively with Ishihara color charts. Ongoing or worsening deficits should prompt ophthalmology evaluation for consideration for a corrective device. Optic filters for red-green color blindness can improve objective test metrics for color contrast sensitivity, but data suggest poor efficacy in improving subjective vision.^73^ Effective optic filter lenses remain elusive for now, but screening for loss of color discrimination may be helpful to stratify the urgency of surgery for patients without any other visual symptoms or deficits.

### Loss of Visual Acuity and Visual Field Loss

Loss of visual acuity and field are among the most debilitating symptoms patients face. Surgery for lesions compressing the optic apparatus usually improves symptoms postoperatively, but the neurosurgeon should be aware of adjuncts and therapies that can accelerate recovery or compensate for ongoing deficits.

There are several techniques and aids to rehabilitate vision, which can commence after the initial postoperative visit if deficits exist. There is evidence from post-stroke visual rehabilitation that visual restorative therapy can improve homonymous visual field loss regardless of age, and its use in optic nerve or chiasmal disorders could also be considered on a case-by-case basis.^74^ Low-vision rehabilitation traditionally focuses on compensation for severe visual loss, and has been reported to improve visual field years after injury.^75,76^ It is particularly effective with biofeedback training on microperimetry.^77^ Time commitment can be a barrier for patients to participate in rehabilitation, for which emerging virtual reality tools might provide additional audiovisual rehabilitation options at home.^78^

For patients with ongoing visual field deficits, prism glasses have been available to expand the visual field for patients with partial or complete homonymous hemianopsia, though their efficacy and clinical adoption are mixed.^79,63^ Prism glasses can be optimized to expand or displace the visual fields as needed. Innovations for monocular visual field deficits, including multiplexed prisms that expand the visual field in one eye, are in development and have yet to be reported for visual deficits from pituitary lesions.^80^ The emerging subspecialty of low vision rehabilitation ophthalmology is a group of specialists at the forefront of addressing these distinctions

### Diplopia

Ongoing postoperative diplopia should be closely evaluated since treatment options differ widely based on its physiologic cause. In hemifield slide, diplopia arises because of insufficient overlapping visual field data points to create a unified visual perception. Diplopia caused by hemifield slide from bitemporal hemianopsia can be improved with prism glasses if there are sufficient unimpaired corresponding bilateral nasal visual field regions.^81^ Urgent referrals to neuro-ophthalmologists and optometrists should be considered if hemifield slide remains on the first postoperative visit.

Steroids can improve diplopia from cranial nerve palsies by reducing inflammatory edema, but do not address underlying compression by the underlying pituitary lesion and have debilitating side effects when used chronically.^32,82,71,83,84,85^ A short course of steroids may be appropriate postoperatively if cranial nerve palsies result from preoperative apoplexy or intraoperative manipulation and irritation. However, the benefit of steroids should be weighed against their impact on confounding the evaluation of the adrenocortical axis.

When diplopia from cranial nerve palsies persists, there is a similar stepwise progression of treatment that often includes attempts at correction with visual therapy, correctional lenses, botulinum toxin, and surgery in severe cases. All patients with postoperative binocular diplopia should be considered for a referral for orthotopic visual therapy. This therapy was developed to treat orbital trauma that causes extraocular muscle damage or palsies and may be helpful for diplopia acquired from tumor compression or iatrogenic damage during surgery. There are several online resources that patients can use to self-administer orthotopic visual therapy that are relatively cost-effective.^86,87^ Correctional lenses can be used concurrently as diplopia improves or as a treatment for fixed diplopia. Prism lenses refract light at specific angles to correct ocular misalignment. They allow the neuro-ophthalmologist to correct diplopia by adjusting hemifields up to 30°C to create overlap between divergent visual fields.^74,65^

The care for debilitating diplopia that persists for weeks to months after surgery should be escalated in conjunction with a neuro-ophthalmology team. Botulinum toxin is often a first line of interventional correction among other injection agents. Botulinum toxin prevents the release of acetylcholine from axonal endings at the neuromuscular junction, causing paralysis of any muscle targeted through injection. When a cranial nerve palsy persists, tonic activation of counteracting muscles causes diplopia. Since cranial nerve palsies produce reliable diplopia syndromes, botulinum toxin injections can weaken or paralyze specific counteracting muscles to bring the globe to a neutral alignment. For instance, botulinum toxin injections in the lateral rectus can help negate the exotropia caused by oculomotor palsy.^70^ Injections to the inferior oblique muscle counteract the effects of a trochlear palsy, and injections to the medial rectus counteract the impact of an abducens palsy.^83,84^

Surgical intervention can improve acquired diplopia from cranial nerve palsies when conservative measures and botulinum toxin injections prove insufficient. Surgical options for diplopia should be considered in conjunction with neuro-ophthalmologists and oculoplastic surgeons and depend on the cadence of symptoms, the specific cranial nerves involved, and the degree of extraocular muscle dysfunction. The goal of surgery for diplopia is usually to re-align the affected eye through muscle and tendon transpositions as well as resective reductions. Given the high rate of recovery from many extraocular movement palsies, waiting 6 months before considering surgery for persistent diplopia maximizes the opportunity for spontaneous recovery.

Surgery for oculomotor palsy includes medial rectus resection and lateral rectus recession to correct horizontal deviation, and superior oblique tendon transposition to adduct the globe into a neutral position.^84,88,89^ After alignment of the affected eye, lid ptosis can also be surgically corrected. The selection of surgery for trochlear nerve palsy depends on the laxity of the superior oblique muscle on forced duction testing and the degree of hypertropia. For cases with minor hypertropias, the inferior oblique can be reduced. For larger hypertropias, inferior oblique reduction can be combined with superior rectus recession.^83^ For partial abducens nerve palsies, the lateral rectus may be resected and the ipsilateral medial rectus is recessed. For complete lateral rectus muscle paresis, there are several complex reconstructions that can be considered.^90^

### Exploring the Neurobiology of Visual Impairments

Despite advances in preoperative and intraoperative testing, a subset of patients have persistent visual symptoms postoperatively. The pathophysiological basis for this remains poorly understood, though it is assumed that a combination of pressure injury and microvascular congestion leads to permanent nerve injury.

The neurobiology of optic nerve injury remains an active area of investigation, and studies have demonstrated several challenges that need to be overcome. Evidence from optic nerve crush models in rodents and mammals has demonstrated that the retinal ganglion cells that comprise the optic nerve have poor regeneration potential after a crush injury.^91,92^ There are several molecular and cellular factors that inhibit regeneration in the optic nerve microenvironment, including immune infiltration, glial scarring, myelin-secreted factors, and the decline of trophic factors with aging.^91,92^ Promoting neural regeneration and preventing inhibition both contribute to optic nerve recovery in various preclinical models, but the development of treatments to restore or regenerate injured RGCs in optic nerves remains at an early stage.^92,93^

Nonetheless, there are several promising lines of inquiry that have demonstrated therapeutic potential in preclinical models. For instance, intraocular injections of trophic factors may promote optic nerve regeneration.^94^ Retinal stem cell transplantation may be applied to regenerate the optic nerve after compressive injuries.^95,96^ Extracellular matrix scaffold injections are also an active area of basic research for optic nerve regeneration, but remain far from in-human clinical testing.^97^ Cortical visual neural prostheses are also in the early stages of development and also hold promise to restore vision in blind patients.^66^ Optic nerve and lateral geniculate electrical stimulation are also being explored, but surgical implantation and tuning of these devices will likely be a barrier to their implementation. It is unclear whether these findings will translate to more subacute or chronic damage caused by extrinsic compression by pituitary lesions. Further studies may help identify better methods to assess cranial nerve dysfunction at a cellular level and identify adjunctive therapies that can promote cranial nerve regeneration.

## Conclusion

Pituitary lesions frequently cause visual deficits that reduce quality of life and may persist after surgery. Standard and emerging methods exist to monitor impending deficits, reduce the risk of injury, and manage deficits postoperatively. These treatments include short-term corticosteroids, monocular occlusion, specialized prism glasses, botulinum injections, and oculoplastic surgery. As the biological underpinnings of damage to optic nerve and cavernous sinus cranial nerves become further elucidated, there may be new medical therapies and devices to improve vision postoperatively. Patients with persistent visual deficits after pituitary surgery should be managed by a multidisciplinary team including neurosurgeons, neuro-ophthalmologists, and oculoplastic surgeons to coordinate individualized treatment.

## References

[1] Al-Mefty O , Holoubi A, Rifai A, Fox JL. Microsurgical removal of suprasellar meningiomas. Neurosurgery. 1985;16(3):364–372. 3982616 10.1227/00006123-198503000-00014

[2] Eskandary H , Sabba M, Khajehpour F, Eskandari M. Incidental findings in brain computed tomography scans of 3000 head trauma patients. Surg Neurol. 2005;63(6):550–3; discussion 553. 15936382 10.1016/j.surneu.2004.07.049

[3] Westmark KD , Kim DH, Riascos RF. Incidental Findings in Neuroimaging and Their Management: A Guide for Radiologists, Neurosurgeons, and Neurologists. Leipzig, Germany: Thieme; 2020.

[4] Ogra S , Nichols AD, Stylli S, et al Visual acuity and pattern of visual field loss at presentation in pituitary adenoma. J Clin Neurosci. 2014;21(5):735–740. 24656736 10.1016/j.jocn.2014.01.005

[5] Briet C , Salenave S, Bonneville JF, Laws ER, Chanson P. Pituitary apoplexy. Endocr Rev. 2015;36(6):622–645. 26414232 10.1210/er.2015-1042

[6] Muthukumar N. Pituitary apoplexy: A comprehensive review. Neurol India. 2020;68(suppl):S72–S78. 32611895 10.4103/0028-3886.287669

[7] Berthold-Lindstedt M , Ygge J, Borg K. Visual dysfunction is underestimated in patients with acquired brain injury. J Rehabil Med. 2017;49(4):327–332. 28350414 10.2340/16501977-2218

[8] van der Aa HPA , Comijs HC, Penninx BWJH, van Rens GHMB, van Nispen RMA. Major depressive and anxiety disorders in visually impaired older adults. Invest Ophthalmol Vis Sci. 2015;56(2):849–854. 25604690 10.1167/iovs.14-15848

[9] McKean-Cowdin R , Varma R, Wu J, Hays RD, Azen SP; Los Angeles Latino Eye Study Group. Severity of visual field loss and health-related quality of life. Am J Ophthalmol. 2007;143(6):1013–1023. 17399676 10.1016/j.ajo.2007.02.022 PMC2731547

[10] Pokorny J. Congenital and Acquired Color Vision Defects. Philadelphia: Saunders; 1979.

[11] Thomé C , Zevgaridis D. Delayed visual deterioration after pituitary surgery--a review introducing the concept of vascular compression of the optic pathways. Acta Neurochir. 2004;146(10):1131–5; discussion 1135. 15744849 10.1007/s00701-004-0331-3

[12] Brown MM , Brown GC, Sharma S, Busbee B, Brown H. Quality of life associated with unilateral and bilateral good vision. Ophthalmology. 2001;108(4):643–7; discussion 647. 11297474 10.1016/s0161-6420(00)00635-7

[13] Brown GC. Vision and quality-of-life. Trans Am Ophthalmol Soc. 1999;97:473–511. 10927011 10.1016/s0002-9394(00)00513-4

[14] Vié AL , Raverot G. Modern neuro-ophthalmological evaluation of patients with pituitary disorders. Best Pract Res Clin Endocrinol Metab. 2019;33(2):101279. 31178379 10.1016/j.beem.2019.05.003

[15] Simunovic MP. Acquired color vision deficiency. Surv Ophthalmol. 2016;61(2):132–155. 26656928 10.1016/j.survophthal.2015.11.004

[16] Poon A , McNeill P, Harper A, O’Day J. Patterns of visual loss associated with pituitary macroadenomas. Aust N Z J Ophthalmol. 1995;23(2):107–115. 7546685 10.1111/j.1442-9071.1995.tb00138.x

[17] Kasputytė R , Slatkevičienė G, Liutkevičienė R, et al Changes of visual functions in patients with pituitary adenoma. Medicina (Kaunas, Lithuania). 2013;49(3):132–137. 23893057

[18] Agosti E , Alexander AY, Pinheiro-Neto CD, et al Letter: Visual field defects in the setting of Suprasellar lesions: Could vascularization patterns of the optic chiasm play a role? Neurosurgery. 2022;91(3):e102–e103. 35876674 10.1227/neu.0000000000002069

[19] Salaud C. Commentary: Letter: Visual field defects in the setting of Suprasellar lesions: Could vascularization patterns of the optic chiasm play a role? Neurosurgery. 2024;94(4):e52. 38265215 10.1227/neu.0000000000002845

[20] Kao LY , Liu CH, Yang ML. Management of diplopia with visual-field defects. Taiwan J Ophthalmol. 2017;7(1):22–27. 29018750 10.4103/tjo.tjo_5_17 PMC5525597

[21] Oda Y , Amano K, Masui K, Kawamata T. Clinical features of pituitary or Parasellar tumor onset with cranial nerve palsy: Surgical intervention considerations. World Neurosurg. 2023;175:e832–e840. 37062334 10.1016/j.wneu.2023.04.031

[22] Saleem QA , Cheema AM, Tahir MA, et al Outcome of unilateral lateral rectus recession and medial rectus resection in primary exotropia. BMC Res Notes. 2013;6:257. 23834953 10.1186/1756-0500-6-257 PMC3708763

[23] Hage R , Eshraghi SR, Oyesiku NM, et al Third, fourth, and sixth cranial nerve palsies in pituitary apoplexy. World Neurosurg. 2016;94:447–452. 27436207 10.1016/j.wneu.2016.07.026 PMC5064865

[24] Perosevic M , Jones PS, Tritos NA. Magnetic resonance imaging of the hypothalamo-pituitary region. Handb Clin Neurol. 2021;179:95–112. 34225987 10.1016/B978-0-12-819975-6.00004-2

[25] Law M , Wang R, Liu CSJ, et al Value of pituitary gland MRI at 7 T in Cushing’s disease and relationship to inferior petrosal sinus sampling: Case report. J Neurosurg. 2018;130(2):1–5. 10.3171/2018.4.JNS171969a 30594120

[26] Al-Louzi O , Prasad S, Mallery RM. Utility of optical coherence tomography in the evaluation of sellar and parasellar mass lesions. Curr Opin Endocrinol Diabetes Obes. 2018;25(4):274–284. 29771751 10.1097/MED.0000000000000415

[27] Kanamori A , Nakamura M, Matsui N, et al Optical coherence tomography detects characteristic retinal nerve fiber layer thickness corresponding to band atrophy of the optic discs. Ophthalmology. 2004;111(12):2278–2283. 15582087 10.1016/j.ophtha.2004.05.035

[28] Parrozzani R , Clementi M, Kotsafti O, et al Optical coherence tomography in the diagnosis of optic pathway gliomas. Invest Ophthalmol Vis Sci. 2013;54(13):8112–8118. 24169000 10.1167/iovs.13-13093

[29] Avery RA , Mansoor A, Idrees R, et al Optic pathway glioma volume predicts retinal axon degeneration in neurofibromatosis type 1. Neurology. 2016;87(23):2403–2407. 27815398 10.1212/WNL.0000000000003402 PMC5177678

[30] Park HH , Oh MC, Kim EH, et al Use of optical coherence tomography to predict visual outcome in parachiasmal meningioma. J Neurosurg. 2015;123(6):1489–1499. 26162035 10.3171/2014.12.JNS141549

[31] Danesh-Meyer HV , Wong A, Papchenko T, et al Optical coherence tomography predicts visual outcome for pituitary tumors. J Clin Neurosci. 2015;22(7):1098–1104. 25891894 10.1016/j.jocn.2015.02.001

[32] Kumar S , Kumar A, Gill MS, Maheshwari V. Optic tract edema: A rare entity in pituitary Macroadenoma. Asian J Neurosurg. 2019;14(1):307–309. 30937062 10.4103/ajns.AJNS_178_18 PMC6417355

[33] Mamelak AN , Little AS, Gardner PA, et al A prospective comparative analysis of surgical versus non-surgical management of pituitary apoplexy: Analysis of The Pituitary Apoplexy Surgical Treatment and Outcomes Registry (PASTOR). 2024;109(2):e711–e725. doi: https://doi.org/10.2139/ssrn.4397805 37698130

[34] Kelly PD , Fernando SJ, Malenke JA, et al The effect of timing of surgery in pituitary apoplexy on continuously valued visual acuity. J Neurol Surg B Skull Base. 2021;82(S 03):e70–e78. 34306919 10.1055/s-0040-1701217 PMC8289513

[35] Outcome in pituitary apoplexy patients, stratified by delay between symptom appearance and surgery: a single center retrospective analysis. Clin Neurol Neurosurg. 2021;210:106991. 34700278 10.1016/j.clineuro.2021.106991

[36] Rutkowski MJ , Kunwar S, Blevins L, Aghi MK. Surgical intervention for pituitary apoplexy: An analysis of functional outcomes. J Neurosurg. 2017;129(2):417–424. 28946177 10.3171/2017.2.JNS1784

[37] Wilson WB , Kirsch WM, Neville H, et al Monitoring of visual function during parasellar surgery. Surg Neurol. 1976;5(6):323–329. https://pubmed.ncbi.nlm.nih.gov/180620/. Accessed October 28, 2023. 180620

[38] Feinsod M , Selhorst JB, Hoyt WF, Wilson CB. Monitoring optic nerve function during craniotomy. J Neurosurg. 1976;44(1):29–31. 1244432 10.3171/jns.1976.44.1.0029

[39] Kodama K , Goto T, Sato A, et al Standard and limitation of intraoperative monitoring of the visual evoked potential. Acta Neurochir. 2010;152(4):643–648. 20127123 10.1007/s00701-010-0600-2

[40] Luo Y , Regli L, Bozinov O, Sarnthein J. Clinical utility and limitations of intraoperative monitoring of visual evoked potentials. PLoS One. 2015;10(3):e0120525. 25803287 10.1371/journal.pone.0120525 PMC4372588

[41] Feng R , Schwartz J, Loewenstern J, et al The predictive role of intraoperative visual evoked potentials in visual improvement after endoscopic pituitary tumor resection in large and complex tumors: Description and validation of a method. World Neurosurg. 2019;126:e136–e143. 30794978 10.1016/j.wneu.2019.01.278

[42] Zhu H , Qiao N, Yang X, et al The clinical application of intraoperative visual evoked potential in recurrent craniopharyngiomas resected by extended endoscopic endonasal surgery. Clin Neurol Neurosurg. 2022;214:107149. 35151969 10.1016/j.clineuro.2022.107149

[43] Jashek-Ahmed F , Cabrilo I, Bal J, et al Intraoperative monitoring of visual evoked potentials in patients undergoing transsphenoidal surgery for pituitary adenoma: A systematic review. BMC Neurol. 2021;21(1):287. 34301198 10.1186/s12883-021-02315-4 PMC8299587

[44] Li ZY , Li MC, Liang JT, et al Usefulness of intraoperative electromyographic monitoring of oculomotor and abducens nerves during skull base surgery. Acta Neurochir. 2017;159(10):1925–1937. 28766024 10.1007/s00701-017-3268-z

[45] Schlake HP , Goldbrunner R, Siebert M, Behr R, Roosen K. Intra-Operative electromyographic monitoring of extra-ocular motor nerves (Nn. III, VI) in skull base surgery. Acta Neurochir. 2001;143(3):251–261. 11460913 10.1007/s007010170105

[46] Gnanalingham KK , Bhattacharjee S, Pennington R, Ng J, Mendoza N. The time course of visual field recovery following transphenoidal surgery for pituitary adenomas: Predictive factors for a good outcome. J Neurol Neurosurg Psychiatry. 2005;76(3):415–419. 15716538 10.1136/jnnp.2004.035576 PMC1739567

[47] Cohen AR , Cooper PR, Kupersmith MJ, Flamm ES, Ransohoff J. Visual recovery after transsphenoidal removal of pituitary adenomas. Neurosurgery. 1985;17(3):446–452. 4047355 10.1227/00006123-198509000-00008

[48] Seuk JW , Kim CH, Yang MS, Cheong JH, Kim JM. Visual outcome after transsphenoidal surgery in patients with pituitary apoplexy. J Korean Neurosurg Soc. 2011;49(6):339–344. 21887391 10.3340/jkns.2011.49.6.339 PMC3158476

[49] Peter M , De Tribolet N. Visual outcome after transsphenoidal surgery for pituitary adenomas. Br J Neurosurg. 1995;9(2):151–157. 7632360 10.1080/02688699550041485

[50] Kerrison JB , Lynn MJ, Baer CA, et al Stages of improvement in visual fields after pituitary tumor resection. Am J Ophthalmol. 2000;130(6):813–820. 11124302 10.1016/s0002-9394(00)00539-0

[51] Chuang CC , Chen E, Huang YC, et al Surgical outcome of oculomotor nerve palsy in pituitary adenoma. J Clin Neurosci. 2011;18(11):1463–1468. 21920756 10.1016/j.jocn.2011.02.041

[52] Wang MTM , King J, Symons RCA, et al Temporal patterns of visual recovery following pituitary tumor resection: a prospective cohort study. J Clin Neurosci. 2021;86:252–259. 33775337 10.1016/j.jocn.2021.01.007

[53] Charles U , Anthony A, Emeka N, et al Visual and endocrine outcome following surgery for pituitary adenoma in a tertiary hospital, North Central Nigeria. Br J Neurosurg. 2022:1–4. 10.1080/02688697.2021.1947973 35481405

[54] Tagoe NN , Essuman VA, Bankah P, et al Visual outcome of patients with pituitary adenomas following surgery and its contributory factors at a tertiary hospital in Ghana. Ethiop J Health Sci. 2019;29(1):895–902. 30700957 10.4314/ejhs.v29i1.11 PMC6341437

[55] Veldman MHJ , van der Aa HPA, Bode C, et al E-nergEYEze, a vision-specific eHealth intervention based on cognitive behavioral therapy and self-management to reduce fatigue in adults with visual impairment: Study protocol for a randomized controlled trial. Trials. 2021;22(1):966. 34963472 10.1186/s13063-021-05935-w PMC8715593

[56] Hernández-Moreno L , Senra H, Moreno N, Macedo AF. Is perceived social support more important than visual acuity for clinical depression and anxiety in patients with age-related macular degeneration and diabetic retinopathy? Clin Rehabil. 2021;35(9):1341–1347. 33657906 10.1177/0269215521997991 PMC8361471

[57] Papadopoulos K , Papakonstantinou D, Montgomery A, Solomou A. Social support and depression of adults with visual impairments. Res Dev Disabil. 2014;35(7):1734–1741. 24679546 10.1016/j.ridd.2014.02.019

[58] Niketeghad S , Pouratian N. Brain machine interfaces for vision restoration: The current state of cortical visual prosthetics. Neurotherapeutics. 2019;16(1):134–143. 30194614 10.1007/s13311-018-0660-1 PMC6361050

[59] Male SR , Shamanna BR, Bhardwaj R, Bhagvati C, Theagarayan B. Color vision devices for color vision deficiency patients: A systematic review and meta-analysis. Health Sci Rep. 2022;5(5):e842. 36189411 10.1002/hsr2.842 PMC9498227

[60] de Haan GA , Melis-Dankers BJM, Brouwer WH, Tucha O, Heutink J. The effects of compensatory scanning training on mobility in patients with homonymous visual field defects: a randomized controlled trial. PLoS One. 2015;10(8):e0134459. 26275160 10.1371/journal.pone.0134459 PMC4537273

[61] Badawy AR , Hassan MU, Elsherif M, et al Contact lenses for color blindness. Adv Healthc Mater. 2018;7(12):e1800152. 29696828 10.1002/adhm.201800152 PMC6691754

[62] Sabel BA , Gao Y, Antal A. Reversibility of visual field defects through induction of brain plasticity: vision restoration, recovery and rehabilitation using alternating current stimulation. Neural Regener Res. 2020;15(10):1799–1806. 10.4103/1673-5374.280302 PMC7513964 32246620

[63] Goodwin D. Homonymous hemianopia: Challenges and solutions. Clin Ophthalmol. 2014;8:1919–1927. 25284978 10.2147/OPTH.S59452 PMC4181645

[64] Mueller I , Mast H, Sabel BA. Recovery of visual field defects: A large clinical observational study using vision restoration therapy. Restor Neurol Neurosci. 2007;25(5-6):563–572. 18334773

[65] Goodlaw E. Rehabilitating a patient with bitemporal hemianopia. Am J Optom Physiol Opt. 1982;59(7):617–619. 7124902 10.1097/00006324-198207000-00011

[66] Chen HY , Tsai RK, Howng SL. Acute painful oculomotor nerve paresis caused by pituitary apoplexy--a case report. Kaohsiung J Med Sci. 1999;15(7):437–440. 10465926

[67] Tamhankar MA , Ying GS, Volpe NJ. Success of prisms in the management of diplopia due to fourth nerve palsy. J Neuroophthalmol. 2011;31(3):206–209. 21378578 10.1097/WNO.0b013e318211daa9 PMC4090702

[68] Talebnejad MR , Tahamtan M, Nowroozzadeh MH. Botulinum toxin injection for treatment of acute traumatic superior oblique muscle palsy. J Ophthalmic Vis Res. 2015;10(3):263–267. 26730311 10.4103/2008-322X.170350 PMC4687259

[69] Yumuşak E , Yolcu U, Küçükevcilioğlu M, Diner O, Mutlu FM. Outcomes of unilateral inferior oblique myectomy surgery in inferior oblique overaction due to superior oblique palsy. Turk J Ophthalmol. 2016;46(1):21–24. 27800253 10.4274/tjo.02170 PMC5076305

[70] Modi P , Arsiwalla T. Cranial nerve III palsy. In: StatPearls [Internet]. Treasure Island, FL: StatPearls Publishing; 2023. 30252368

[71] Sanz PM , Escribano J, Gómez de Liaño P, Yela R. Surgical treatment of superior oblique palsy: predictors of outcome. Indian J Ophthalmol. 2017;65(8):723–728. 28820159 10.4103/ijo.IJO_699_16 PMC5598184

[72] Azarmina M , Azarmina H. The six syndromes of the sixth cranial nerve. J Ophthalmic Vis Res. 2013;8(2):160–171. 23943691 PMC3740468

[73] Gómez-Robledo L , Valero EM, Huertas R, Martínez-Domingo MA, Hernández-Andrés J. Do EnChroma glasses improve color vision for colorblind subjects? Opt Express. 2018;26(22):28693–28703. 30470042 10.1364/OE.26.028693

[74] Dundon NM , Bertini C, Làdavas E, Sabel BA, Gall C. Visual rehabilitation: visual scanning, multisensory stimulation and vision restoration trainings. Front Behav Neurosci. 2015;9:192. 26283935 10.3389/fnbeh.2015.00192 PMC4515568

[75] Wiley ZC , Bhat N, Bindiganavile SH, et al Significant visual improvement with vision rehabilitation delayed three decades from disease onset. Am J Ophthalmol Case Rep. 2020;20:100973. 33195878 10.1016/j.ajoc.2020.100973 PMC7642766

[76] van Nispen RM , Virgili G, Hoeben M, et al Low vision rehabilitation for better quality of life in visually impaired adults. Cochrane Database Syst Rev. 2020;1(1):CD006543. 31985055 10.1002/14651858.CD006543.pub2 PMC6984642

[77] Misawa M , Pyatova Y, Sen A, et al Innovative vision rehabilitation method for hemianopsia: comparing pre- and post audio-luminous biofeedback training for ocular motility improving visual functions and quality of life. Front Neurol. 2023;14:1151736. 37114220 10.3389/fneur.2023.1151736 PMC10126773

[78] Daibert-Nido M , Pyatova Y, Cheung K, et al Case report: visual rehabilitation in hemianopia patients. Home-based visual rehabilitation in patients with hemianopia consecutive to brain tumor treatment: feasibility and potential effectiveness. Front Neurol. 2021;12:680211. 34354660 10.3389/fneur.2021.680211 PMC8333276

[79] Rowe FJ , Conroy EJ, Bedson E, et al A pilot randomized controlled trial comparing effectiveness of prism glasses, visual search training and standard care in hemianopia. Acta Neurol Scand. 2017;136(4):551–553. 28028819 10.1111/ane.12725

[80] Jung JH , Castle R, Kurukuti NM, Manda S, Peli E. Field expansion with multiplexing prism glasses improves pedestrian detection for acquired monocular vision. Transl Vis Sci Technol. 2020;9(8):35. 10.1167/tvst.9.8.35 PMC7422757 32855881

[81] Peli E , Satgunam P. Bitemporal hemianopia; its unique binocular complexities and a novel remedy. Ophthalmic Physiol Opt. 2014;34(2):233–242. 24588535 10.1111/opo.12118 PMC3947624

[82] Santos AR , Bello CT, Sousa A, Duarte JS, Campos L. Pituitary apoplexy following systemic anticoagulation. Eur J Case Rep Intern Med. 2019;6(12):001254. 31893198 10.12890/2019_001254 PMC6936922

[83] Khanam S , Sood G. Trochlear nerve palsy. In: StatPearls [Internet]. Treasure Island, FL: StatPearls Publishing; 2023. 33351409

[84] Graham C , Gurnani B, Mohseni M. Abducens nerve palsy. In: StatPearls [Internet]. Treasure Island, FL: StatPearls Publishing; 2023. 29489275

[85] Yasir M , Goyal A, Sonthalia S. Corticosteroid adverse effects. In: StatPearls [Internet]. Treasure Island, FL: StatPearls Publishing; 2023. 30285357

[86] Shainberg MJ. Vision therapy and orthoptics. Am Orthopt J. 2010;60:28–32. 21061881 10.3368/aoj.60.1.28

[87] De Ruiter BJ , Kotha VS, Peiffer AJ, et al Orthoptic vision therapy: Establishing a protocol for management of diplopia following orbital fracture repair. J Craniofac Surg. 2021;32(3):1025–1028. 32969940 10.1097/SCS.0000000000007099

[88] Singh A , Bahuguna C, Nagpal R, Kumar B. Surgical management of third nerve palsy. Oman J Ophthalmol. 2016;9(2):80–86. 27433033 10.4103/0974-620X.184509 PMC4932800

[89] Kattleman B , Flanders M, Wise J. Supramaximal horizontal rectus surgery in the management of third and sixth nerve palsy. Can J Ophthalmol. 1986;21(6):227–230. 3779510

[90] Couser NL , Lenhart PD, Hutchinson AK. Augmented Hummelsheim procedure to treat complete abducens nerve palsy. J AAPOS. 2012;16(4):331–335. 22929448 10.1016/j.jaapos.2012.02.015 PMC4143379

[91] Benowitz LI , Yin Y. Optic Nerve Regeneration. Arch Ophthalmol. 2010;128(8):1059–1064. 20697009 10.1001/archophthalmol.2010.152 PMC3072887

[92] Williams PR , Benowitz LI, Goldberg JL, He Z. Axon regeneration in the mammalian optic nerve. Annu Rev Vis Sci. 2020;6:195–213. 32936739 10.1146/annurev-vision-022720-094953

[93] Zhang S , Zhu H, Pan Y, et al Exploration of the strategies to enhance the regeneration of the optic nerve. Exp Eye Res. 2022;219:109068. 35398207 10.1016/j.exer.2022.109068

[94] Fague L , Liu YA, Marsh-Armstrong N. The basic science of optic nerve regeneration. Ann Transl Med. 2021;9(15):1276. 34532413 10.21037/atm-20-5351 PMC8421956

[95] Tomczak W , Winkler-Lach W, Tomczyk-Socha M, Misiuk-Hojło M. Advancements in ocular regenerative therapies. Biology. 2023;12(5):737. 37237549 10.3390/biology12050737 PMC10215726

[96] da Silva-Junior AJ , Mesentier-Louro LA, Nascimento-Dos-Santos G, et al Human mesenchymal stem cell therapy promotes retinal ganglion cell survival and target reconnection after optic nerve crush in adult rats. Stem Cell Res Ther. 2021;12(1):69. 33468246 10.1186/s13287-020-02130-7 PMC7814601

[97] Ren T , van der Merwe Y, Steketee MB. Developing extracellular matrix technology to treat retinal or optic nerve injury(1,2,3). eNeuro. 2015;2(5):ENEURO.0077–ENEU15.2015. 10.1523/ENEURO.0077-15.2015 PMC4603254 26478910

